# Correction: Spatiotemporal Patterns and Predictability of Cyberattacks

**DOI:** 10.1371/journal.pone.0131501

**Published:** 2015-06-22

**Authors:** Yu-Zhong Chen, Zi-Gang Huang, Shouhuai Xu, Ying-Cheng Lai

The following information is missing from the Funding section: This study was also supported by the NSF of China, Grant No. 11275003.
